# Systematic Reviews as Part of Doctoral Theses and for the Promotion to Associate Professor: A Descriptive Study of University Policies in Sweden

**DOI:** 10.1002/cesm.70069

**Published:** 2026-01-14

**Authors:** Martin Ringsten, Lea Styrmisdottir, Matilda Naesström, Minna Johansson, Matteo Bruschettini, Susanna M. Wallerstedt

**Affiliations:** ^1^ Department of Research, Development, Education and Innovation, Cochrane Sweden, Skåne University Hospital Lund University Lund Sweden; ^2^ Department of Clinical Sciences, Psychiatry Umeå University Umeå Sweden; ^3^ School of Public Health and Community Medicine, Global Center for Sustainable Healthcare, Sahlgrenska Academy University of Gothenburg Gothenburg Sweden; ^4^ Department of Pharmacology, Sahlgrenska Academy University of Gothenburg Gothenburg Sweden; ^5^ Center for Health Technology Assessment, Sahlgrenska University Hospital Gothenburg Region Västra Götaland Sweden

## Abstract

**Background:**

Almost a decade ago, about half of biomedical PhD programs across Europe specifically stated that systematic reviews could not be accepted as part of a doctoral thesis, illustrating limited merit value at that time. The aim of this study was to explore current Swedish university policies on this research design.

**Methods:**

Policy documents for PhD theses and applications to associate professor positions were obtained from all medical faculties at universities in Sweden. Instructions regarding systematic reviews, with focus on their merit value and related aspects, were independently extracted and categorized by two authors, with discrepancies resolved in consensus discussions.

**Results:**

All seven medical faculties accepted at least one systematic review within a PhD thesis, five restricted the number of such studies accepted, and five provided instructions regarding this study design. Regarding policies for promotion to associate professor, six medical faculties accepted at least one published systematic review to merit recognition―the remaining one required meta‐analyses for acceptance―and three explicitly restricted the number of systematic reviews. No restrictions or guidance were provided for other designs intended to answer specific research questions.

**Conclusion:**

As of 2025, systematic reviews appear to be generally recognized as contributing to authors' academic merit. For this research design exclusively, some universities impose restrictions that may limit their recognition, and some provide guidance which may help ensure quality in reporting. These findings may encourage research to evaluate the merit value of systematic reviews in other settings, and to examine potential implications of restrictions and guidance in policy documents.

## 1 Introduction

A systematic review is a study design that aims to find, assess and summarize all the empirical evidence that fulfills pre‐specified eligibility criteria to answer a specific research question [1]. The number of publications using this design within medicine and health sciences has increased considerably since the 1990s [2], and the methods and approach have continuously been updated and refined over the years [3, 4].

When properly conduced, a systematic review provides a transparent picture of the overall state of evidence. It therefore informs further research needs and can also constitute a basis for evidence‐based decision‐making in healthcare. However, methodological limitations in the design and execution of studies claiming to use this study design are common [2, 5, 6].

Two major milestones in the academic context are the Doctor of Philosophy (PhD) degree as well as the promotion to associate professor, i.e., being acknowledged as an independent researcher. In 2016, the majority of universities running biomedical PhD programs, across 68 institutions in 37 countries stated that this design was not accepted as part of a PhD thesis [7]. As far as we know, the current status of the merit value of conducting systematic reviews from a university perspective, is not known.

## 2 Objective

To investigate the academic merit value of conducting systematic reviews by exploring their acceptance within PhD theses and their merit contributions in applications for promotion to associate professor.

## 3 Materials and Methods

### 3.1 Setting

In Sweden, the PhD degree is regulated in the Swedish Higher Education Ordinance Act, whereas no national regulations exist regarding the title of associate professor. To obtain a PhD degree, 10 national outcomes must be met (available in the Appendix, Table S1). Based on this framework, universities develop local policies to guide PhD students, supervisors, and researchers.

### 3.2 Study Design and Data Collection

In this descriptive study, inspired by the framework used in document analysis in policy research [8], we retrieved official policy documents related to acquiring a PhD degree and for the promotion to associate professor. M. R. searched the websites of all universities in Sweden with focus on their medical faculty (University of Gothenburg (GU), Karolinska Institute (KI), Linköping University (LiU), Lund University (LU), Örebro University (ORU), Umeå University (UmU), and Uppsala University (UU)) for documents regarding these policies. The documents had to clearly identify an official sender from a recognized part of the university (such as a research studies committee or a faculty board) and specify an effective date that did not conflict with any subsequent updates. Documents available in Swedish were prioritized over English translations.

When a policy was not identified or required employee access, we reached out within our networks to identify and get access to the documents. The validity of the documents was checked by reaching out to colleagues within each university for confirmation. An updated search was conducted on February 12, 2025 to capture potential updates.

### 3.3 Data Extraction and Analysis

Instructions regarding systematic reviews, with focus on their merit value and related aspects, and corresponding information for a PhD degree and the promotion to associate professor in general, were independently extracted and categorized, by two authors (M. R. and either M. N., or L. S.), with discrepancies resolved in consensus discussions. Beforehand, a data extraction form was constructed based on questions outlined in a protocol [9] aiming to identify whether systematic reviews were accepted and potential definitions and restrictions that were applied. We recorded whether other study designs were mentioned correspondingly. The form was piloted on policies for a PhD degree and the promotion to associate professor at LU, followed by updated phrasings and additional guidance for data extraction. The final extraction form included 17 sections, including general information and requirements, systematic review‐focused requirements for a PhD degree and for the promotion to associate professor, respectively, plus comments for clarification. Each section had an accompanying part where the exact sentences related to systematic reviews were extracted. The full form is available in an open repository [9].

## 4 Results

Local policies for PhD theses and applications for associate professor were available on the websites of six universities, and one had to be accessed from our network (UmU). General policy instructions regarding the acquisition of a PhD degree and for the promotion to associate professor are provided in Table S2.

Details about the university policies regarding systematic reviews are described in Table 1. No definitions or restrictions within all policies were applied to study designs other than systematic reviews.

**Table 1:** Policies regarding systematic reviews within a PhD thesis and for the promotion to associate professor in all medical faculties at universities in Sweden.

		GU	KI	LiU	LU	ORU	UmU	UU
Definition of a systematic review provided	PhD	No	Yes	Yes	No	No	No	No
	Ass Prof	No	No	No	No	No	No	No
At least one systematic review accepted	PhD	Yes	Yes	Yes	Yes	Yes	Yes	Yes
	Ass Prof	Yes	Yes	Yes	Yes	Yes	Yes	(Yes^a^)
Other types of review designs accepted among required papers	PhD	No^b^	Not defined	Not defined	Not defined	Not defined	Not defined	No^c^
	Ass Prof	Not defined	No	No	No	No	Not defined	No
Methodological restrictions for systematic reviews	PhD	Yes	Yes	Yes	Yes	No	No	Yes
	Ass Prof	No	No	No	No	No	No	Yes
Numerical restrictions of systematic reviews	PhD	No	No	Yes	Yes	Yes	Yes	No
	Ass Prof	No	No	Yes	Yes	Yes	No	No
If restricted, how many are accepted	PhD	NA	NA	1	All studies except one	1	1	NA
	Ass Prof	NA	NA	“Occasional”	5	“Occasional”	NA	NA
Author order restrictions	PhD	No	No	Yes	No	No	No	No
	Ass Prof	No	No	Yes	No	No	No	No
Restrictions also for other study designs	PhD	No	No	No	No	No	No	No
	Ass Prof	NA	NA	No	No	No	NA	NA

Abbreviations: Ass Prof = associate professor, GU = University of Gothenburg, KI = Karolinska Institute, LiU = Linköping University, LU = Lund University, ORU = Örebro University, UU = Uppsala University, UmU = Umeå University

^a^ Meta‐analysis.

^b^ “Conventional review” (not defined) explicitly not accepted.

^c^ “Common review” (not defined) explicitly not accepted.

Regarding the acquisition of a PhD degree, all universities accepted at least one systematic review, and two provided a definition of this design by giving examples of what to be considered a systematic review (KI and LiU). Other types of reviews were either not mentioned, described ambiguously, or explicitly not accepted. Three universities did not restrict the number of systematic reviews accepted within a PhD thesis (GU, KI and UU), and three explicitly restricted the maximum to one (LiU, ORU and UmU) or all but one (LU). Five universities included instructions in their policies, with statements like “thorough analysis,” “common practice,” or “established methods” (GU, LU, ORU, UU, and UmU), whereas two referred to reporting guidelines (KI and LiU).

Regarding the promotion to associate professor, six universities accepted at least one systematic review (GU, KI, LiU, LU, ORU, and UmU), and the remaining university required meta‐analyses (UU). None of the universities provided definitions of a systematic review. Five universities restricted other review designs like “overview articles,” “reviews,” or “non‐systematic reviews” (KI, LiU, LU, ORU, and UU). Four universities did not restrict the number of systematic reviews that held merit value (GU, KI, UmU and UU), two accepted “occasional” systematic reviews (LiU and ORU), and one accepted up to five systematic reviews (LU).

## 5 Discussion

As of 2025, systematic reviews appear to be generally recognized as contributing to authors' academic merit; all universities accepted at least one systematic review within a PhD thesis, and if a restriction to meta‐analyses is interpreted to correspond to a systematic review, they all accepted at least one published systematic review for merit recognition in promotion to associate professor. Some universities, however, limit the number of systematic reviews to be accounted for. As the knowledge of key characteristics of the systematic review design has been shown to vary among those in charge of European PhD programs, with almost half of them stating that systematic reviews must contain meta‐analyses in 2016 [7], one may speculate that increased knowledge may have contributed to policy development over the last decade. Nevertheless, our finding that some universities provided vague information on what constitutes an acceptable systematic review may indicate that the design remains, to some extent, unclear for parts of the research community.

As a systematic review can be considered a key design to attain key outcomes for a PhD degree—such as developing a broad and in‐depth understanding of the research field, as well as the ability to conduct scientific analysis, synthesis and critical review—our results are encouraging. Some university policies, however, restricted the number of systematic reviews that could count toward potential supervisors' academic merit. Exploring underlying reasons for restrictions as well as potential implications would therefore be of interest—especially since this design helps identify knowledge gaps and provides a foundation for evidence‐based decision‐making in healthcare.

Some university policies included guidance specifically targeting this design. This may illustrate attempts to help mitigate the conduct of systematic reviews that do not meet sufficient quality standards [5]. The value of providing guidance in policies could also be worth further investigation. Nevertheless, it must be noted that the instructions could be more precise. For instance, the PRISMA guidelines provide information regarding the reporting of a systematic review [10] and not the conduct. Furthermore, with regard to the quality of research conduct, it should be acknowledged that guidance may also be relevant for study designs other than systematic reviews.

A strength of the present study is that it provides a detailed analysis of university policies regarding merit recognition of the systematic review design in the academic context. Other strengths include that we followed a prespecified protocol; conducted independent extraction followed by consensus discussions; and used the official policy documents as our source of data. In addition, we share our piloted forms, data set and policy documents for other researchers to build upon our work, and provide suggestions for further research.

An obvious limitation is that our analyses are restricted to the Swedish setting. Another limitation is the focus on systematic reviews. However, other review types based on systematic literature searches were not mentioned in the policies, such as scoping and mapping reviews as well as umbrella reviews where methodological development is still rapidly evolving [11, 12, 13]. Indeed, some studies included in Swedish PhD theses in 2021 described as systematic reviews were in fact broader review types and not systematic reviews [5].

In conclusion, systematic reviews now generally appear to be recognized as contributing to authors' academic merit, while some universities impose restrictions exclusively for this study design. Future research could aim to map the merit value of systematic reviews in other settings, its development over time, and examine the potential implications of imposing restrictions and offering guidance in policy documents.

## Author Contributions

**Martin Ringsten:** conceptualization, investigation, formal analysis, methodology, data curation, writing – original draft, writing – review and editing, project administration. **Lea Styrmisdottir:** investigation, writing – review and editing. **Mathilda Naesström:** investigation, writing – review and editing. **Minna Johansson:** conceptualization, methodology, writing – review and editing. **Matteo Bruschettini:** conceptualization, methodology, writing – review and editing. **Susanna Wallerstedt:** conceptualization, formal analysis, writing – original draft, writing – review and editing.

## Funding

The authors received no specific funding for this work.

## Conflicts of Interest

Martin Ringsten performs systematic reviews and deliver training related to systematic reviews as part of his employment at Cochrane Sweden. He declares no other conflict of interest. Lea Styrmisdottir performs systematic reviews as part of her role at Cochrane Sweden. She declares no other conflicts of interest. Mathilda Naesström performs systematic reviews as part of her employments at the University of Umeå and teaches medical students as well as healthcare professionals within the field. She declares no other conflicts of interest. Minna Johansson performs systematic reviews as part of her role at Cochrane Sweden. She declares no other conflicts of interest. Matteo Bruschettini performs systematic reviews and deliver training related to systematic reviews as part of his employment at Cochrane Sweden. He declares no other conflict of interest. Susanna Wallerstedt performs systematic reviews as part of her employments at the University of Gothenburg and at the Sahlgrenska University Hospital, and teaches medical students as well as healthcare professionals within the field. She declares no other conflicts of interest.

## Supporting information


**Table S1:** Learning outcomes for doctoral students in Sweden, as stated in the Higher Education Ordinance, Annex 2. **Table S2:** General policies for a PhD degree and promotion to associate professor at all Swedish universities.

## Data Availability

The data that support the findings of this study are openly available in Open Science Framework at https://osf.io/fduj7/overview. A protocol was registered a priori at Open Science Framework at https://osf.io/fduj7/.
