# Supplementary material for: Systematic Reviews as Part of Doctoral Theses and for the Promotion to Associate Professor: A Descriptive Study of University Policies in Sweden
Source: Cochrane Evid Synth Methods. 2026 Jan 14;4(1):e70069. doi: 10.1002/cesm.70069 (PMC12806540; doi:10.1002/cesm.70069)
Supplement: Supplementary file 1 — Table S1: Learning outcomes for doctoral students in Sweden, as stated in the Higher Education Ordinance, Annex 2. Table S2: General policies for a PhD degree and promotion to associate professor at all Swedish universities. [file CESM-4-e70069-s001.docx]

**Appendix**

**Deviations from study protocol**

We initially planned to verify each identified policy document with the official university office, but abandoned this approach after a pilot yielded no response. Instead, we confirmed the correctness of the documents with colleagues at the respective universities. We also intended to investigate the development over time, by including policies from 2016 to 2024; however, this part was omitted due to feasibility concerns following the unsuccessful pilot. Finally, to map the context, we extracted information on general policies, such as the total number of required studies and other restrictions.

**Table S1** Learning outcomes for doctoral students in Sweden, as stated in the Higher Education Ordinance, Annex 2.

| For the Degree of Doctor, the third-cycle student shall: | |
| --- | --- |
| *Knowledge and understanding* | - demonstrate broad knowledge and systematic understanding of the research field as well as advanced and up-to-date specialised knowledge in a limited area of this field, and |
|  | - demonstrate familiarity with research methodology in general and the methods of the specific field of research in particular. |
| *Competence and skills* | - demonstrate the capacity for scholarly analysis and synthesis as well as to review and assess new and complex phenomena, issues and situations autonomously and critically, |
|  | - demonstrate the ability to identify and formulate issues with scholarly precision critically, autonomously and creatively, and to plan and use appropriate methods to undertake research and other qualified tasks within predetermined time frames and to review and evaluate such work, |
|  | - demonstrate through a dissertation the ability to make a significant contribution to the formation of knowledge through his or her own research, |
|  | - demonstrate the ability in both national and international contexts to present and discuss research and research findings authoritatively in speech and writing and in dialogue with the academic community and society in general, |
|  | - demonstrate the ability to identify the need for further knowledge and |
|  | - demonstrate the capacity to contribute to social development and support the learning of others both through research and education and in some other qualified professional capacity. |
| *Judgement and approach* | - demonstrate intellectual autonomy and disciplinary rectitude as well as the ability to make assessments of research ethics, and |
|  | - demonstrate specialised insight into the possibilities and limitations of research, its role in society and the responsibility of the individual for how it is used. |

**Table S2:** General policies for a PhD degree and promotion to associate professor at all Swedish universities. ‘

|  |  | **GU** | **KI** | **LiU** | **LU** | **ORU** | **UmU** | **UU** |
| --- | --- | --- | --- | --- | --- | --- | --- | --- |
| PhD degree | Minimal number of included articles in thesis | 2 | Not defined | 2 | 3 | 3 | 3 | Not defined |
|  | Number of accepted or published articles | 1 | 2 | 2 | 1 | 2 | 2 | 0 |
|  | Number of articles as first author | 1 | 1 | 1 | 1 | 3 | 1 | Half  (less has to be explicitly motivated) |
|  | Maximum number of articles permitted to be conducted before enrolment | Half or less | Not defined | Half or less | 1 | Not defined | Half or less | 1 |
| Associate professor | Number of accepted or published articles | 14 | 15 | 15 | 15 | 15 | 15 | 15 |
|  | Number of articles as first author | 0‒2  (two as either first or last author) | 0‒1  (zero or one as first author, depending on the number of publications as last author) | 0‒2  (two as either first or last author) | 0‒1  (zero or one as first author, depending on the number of publications as last author) | 3‒5  (as either first or last author) | 0‒1  (zero or one as first author, depending on the number of publications as last author) | 0‒3  (as either first or last author) |
|  | Number of articles to be conducted after the PhD | 8^1^ | 8^1^ | 8^1^ | 10 | 8^1^ | 8^1^ | 8^1^ |
|  | Number of articles as last author | 0‒2  (two as either first or last author) | 1‒2  (one, or two if not first author on any other study) | 0‒2  (two as either first or last author) | 1‒2  (one, or two if not first author on any other study) | 3‒5  (as either first or last author) | 1‒2  (one, two if not first author on any other study) | 0‒3  (as either first or last author) |
|  | Restrictions in publishing with previous supervisors | Yes | Yes | Yes | Yes | Yes | Yes | Yes |

GU = University of Gothenburg, Sahlgrenska Academy, KI = Karolinska Institute, LiU = Linköping University, Faculty of Medicine; LU = Lund University, Faculty of Medicine; ORU = Örebro University, Faculty of Medicine. UU = Uppsala University, Faculty of Medicine; UmU = Umeå University, Faculty of Medicine

^1^Described as majority/at least half/essential/considerable, translated to a minimum requirement of eight studies
